# Effect of Lycopene Supplementation on Some Cardiovascular Risk Factors and Markers of Endothelial Function in Iranian Patients with Ischemic Heart Failure: A Randomized Clinical Trial

**DOI:** 10.1155/2022/2610145

**Published:** 2022-10-28

**Authors:** Bahareh Karimian, Azam Soleimani, Ghasem Mohammadsharifi, Kiyan Heshmat-Ghahdarijani, Laila Rejali, Davood Shafie, Atefeh Amerizadeh, Masoumeh Sadeghi

**Affiliations:** ^1^Chamran Cardiovascular Medical and Research Center, Isfahan University of Medical Sciences, Isfahan, Iran; ^2^Department of Orthopedics, Isfahan University of Medical Sciences, Isfahan, Iran; ^3^Heart Failure Research Center, Cardiovascular Research Institute, Isfahan University of Medical Sciences, Isfahan, Iran; ^4^Department of Biochemistry, Islamic Azad University, Falavarjan Branch, Isfahan, Iran; ^5^Cardiac Rehabilitation Research Center, Isfahan Cardiovascular Research Institute, Isfahan University of Medical Sciences, Isfahan, Iran

## Abstract

**Aim:**

This study aimed to explore if supplementary lycopene tablets may help heart failure (HF) patients improve their lipid profile, BP, and the flow-mediated dilation (FMD) index for endothelial function.

**Methods:**

Fifty patients with ischemic HF with a reduced ejection fraction (HFrEF) were randomly assigned to one of two groups: the lycopene group which received 25 mg lycopene tablets once a day for 8 weeks and the control group which received placebo tablets containing starch once a day for 8 weeks.

**Results:**

Our results showed that after two months, the amount of triglyceride (TG) and FMD improved significantly compared to the control, TG decreased (219.27 vs. 234.24), and the mean of FMD increased (5.68 vs. 2.95). Other variables, including total cholesterol (TC), low-density lipoprotein cholesterol (LDL-C), high-density cholesterol (HDL-C), systolic blood pressure (SBP), and diastolic blood pressure (DBP), showed no improvement. Also, only SBP and FMD showed intragroup improvement in the intervention group. In the intervention group, only SBP and FMD exhibited intragroup improvement.

**Conclusions:**

It can be concluded that supplementing with lycopene can enhance endothelial function and reduce the TG levels in ischemic HFrEF patients. However, it had no positive effect on BP, TC, LDL-C, or HDL-C. *Trial Registration*. This clinical trial was registered at the Iranian Registry of Clinical Trials with IRCT registration number: IRCT20210614051574N4.

## 1. Introduction

Heart failure (HF) is a worldwide pandemic that is associated with high morbidity and mortality and becoming more prevalent despite significant advancements in treatments and prevention strategies due to the enormous economic burden [1, 2]. Numerous risk factors have been identified that both predict the incidence of HF and its severity, including hypertension, obesity, smoking, dyslipidemia, and diabetes [3–5]. Dyslipidemia, identified as increased low-density lipoprotein cholesterol (LDL-C), total cholesterol (TC), triglyceride (TG) levels, and/or decreased high-density lipoprotein cholesterol (HDL-C), is found to be associated with decreased systolic and diastolic left ventricular function and increased risk of cardiovascular disease (CVD) [6–9]. In an Iranian study, the TC baseline level was shown to be significantly associated with the risk of MI in men [10]. Long-term hypertension (HTN) can lead to structural and functional changes in patients with HF, such as concentric and eccentric hypertrophy, left ventricular fibrosis, and cardiac remodeling [11]. Oxidative stress, nitric oxide (NO) dysregulation, and endothelial dysfunction play an essential role in the deterioration of HF. Recent clinical trials suggest that improving endothelial function in patients with HF concurrently leads to improvement of heart biomechanics [12].

According to previous reports, diets high in fruits and vegetables, such as a “Mediterranean diet,” are linked to a lower risk of CVD, especially in conjunction with effective secondary prevention medication [13]. Lycopene is a natural red carotenoid pigment found in tomatoes, watermelon, pink grapefruit, guava, papaya, and other fruits [14–16]. Those with higher lycopene concentrations in their adipose tissue found to have a decreased intimal wall thickness and a lower chance of MI [3]. Lycopene may help refine vascular function and prevent CVD in primary or secondary preventative stages [4]. There is a growing body of scientific evidence that the antioxidant properties of lycopene confer resistance to CVD, diabetes, and inflammatory diseases [17]. The number of clinical trial studies that have examined the effect of lycopene supplementation specifically in ischemic HF patients with a reduced ejection fraction (HFrEF) is limited. Hence, the current clinical trial sought to investigate the effect of supplemental lycopene tablets on some CVD risk factors such as lipid profile, blood pressure (BP), and flow-mediated dilation (FMD) as an index of endothelial function in Iranian HFrEF patients.

## 2. Material and Methods

### 2.1. Study Design

**Figure 1:**
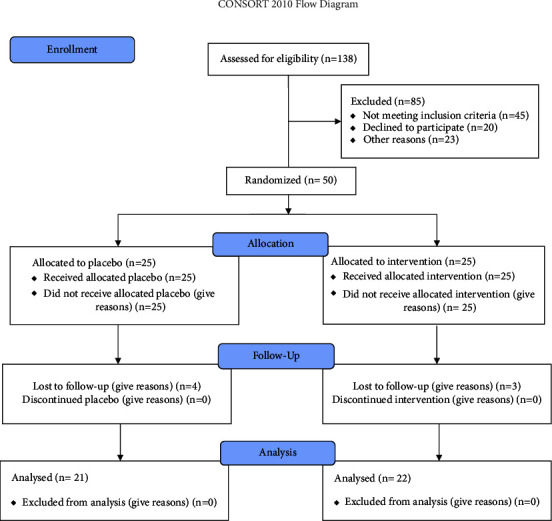
Flow diagram of subjects throughout the study.

The study was conducted at the Chamran Hospital, Isfahan, Iran. The protocol was approved by the local ethics committee of Isfahan University of Medical Sciences. This clinical trial was registered at Iranian Registry of Clinical Trials with IRCT registration number IRCT20210614051574N4. Plans and goals were clearly explained to patients who agreed to participate in this study. They ensured confidentiality of the information, and all participants signed informed consent. They were also assured that participation in the clinical trial would not interfere with their current medication use.  Figure 1 shows the flow diagram of subjects throughout the study (CONSORT 2010 flow diagram).

### 2.2. Inclusion/Exclusion Criteria

The included population was men aged >40 years with HFrEF, according to the American Heart Association guidelines [18].

Inclusion criteria were the agreement of patients to participate in the project, HF due to MI with an ejection fraction below 40% and having a BMI higher than 18.5 and lower than 30.

Exclusion criteria were as follows: suffering from autoimmune diseases, respiratory diseases, acute phase of cancer or surgery, infectious and liver diseases, diabetes mellitus, having other heart diseases, taking corticosteroid or immunosuppressive drugs, and consumption of supplements with antioxidant properties.

### 2.3. Intervention

According to the inclusion criteria, 50 patients with HFrEF were randomized to either the lycopene group, including 25 patients as an intervention group and 25 patients as a control group. The intervention group received lycopene tablets 25 mg made by the 21^st^ Century company once a day for 8 weeks. The control group received placebo tablets containing starch in the same manner. Variables were measured before and after taking medication. A checklist of personal and pharmaceutical information (medical and pharmaceutical history and tobacco use) was created for each person. Anthropometric characteristics such as height, weight, and BMI were measured and recorded on a checklist. All patients underwent high-resolution ultrasonography to record the exact FMD of each patient. FMD of the brachial artery was performed by one investigator throughout the study. The evaluation was performed using a 7 MHz high-resolution ultrasound machine. Participants fasted for at least 8–12 hours before the study. The participants were instructed to abstain from caffeine, high-fat foods, and vitamin C for at least 4–6 hours before the study. At least 10 minutes before scanning, the right brachial artery was fully explored and visualized 10–15 cm above the antecubital fossa. During imaging, anatomical landmarks such as veins and fascia are marked to help maintain the same image of the artery throughout the study. The brachial artery diameter was measured on longitudinal images visualizing the boundaries of the lumen at the proximal (anterior) and distal (posterior) walls. Consequently, the cuff was inflated to 50 mmHg above systolic blood pressure (SBP) to create arterial occlusion and deflated after 5 minutes. FMD was calculated using the vessel lumen diameter 1 min after cuff contraction. FMD was expressed as the change in diameter after stimulation as a percentage of the baseline diameter. The patient's blood pressure is measured three times on the right arm, while that of the seated patient after 15 min of rest. After 5 minutes of rest, the average measured values are recorded.

### 2.4. Blood Collection and Lipid Profile Evaluation

Blood was taken in the morning from the arm veins of patients who had fasted the night before. Centrifugation was used to separate the serum from this, and aliquots were kept at −80°C until the test. TG and TC levels were measured using an enzymatic colorimetric assay (Pars Azmoon kit, Tehran, Iran) by using an autoanalyzer (BT 4500, Biotecnica Instruments, Italy). LDL-C and HDL-C levels were measured directly by the immunoturbidimetric assay (Pars Azmoon kit; Tehran, Iran).

### 2.5. Statistical Analysis

Since the number of samples in both groups was less than 30 and also the test showed that the distribution of all variables was not normal, to compare the variables before and after the intervention between the two groups (drug and placebo) and in each group separately, the Mann–Whitney and Kruskal–Wallis tests were used, respectively. A *P* value less than 0.05 was considered significant.

## 3. Results

**Figure 2:**
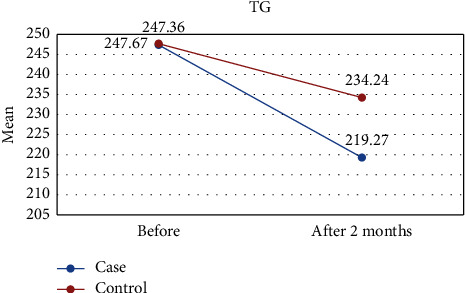
Effect of lycopene supplementation on TG levels.

**Figure 3:**
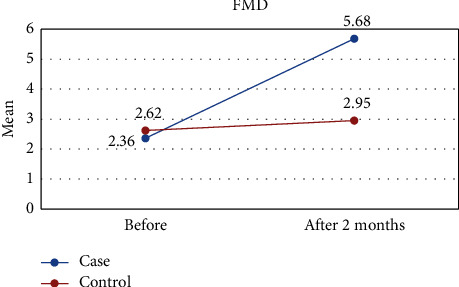
Effect of lycopene supplementation on FMD.

**Figure 4:**
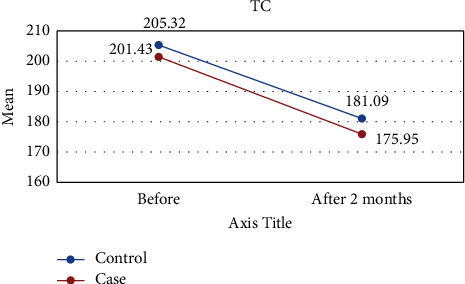
Effect of lycopene supplementation on TC levels.

**Figure 5:**
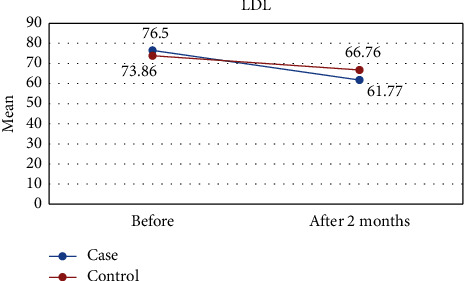
Effect of lycopene supplementation on LDL-C levels.

**Figure 6:**
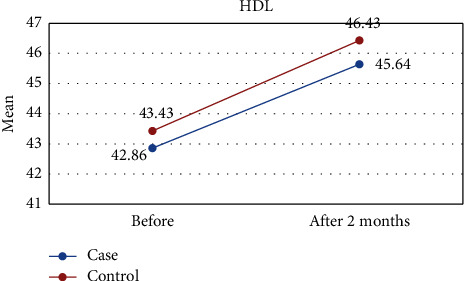
Effect of lycopene supplementation on HDL-C levels.

**Figure 7:**
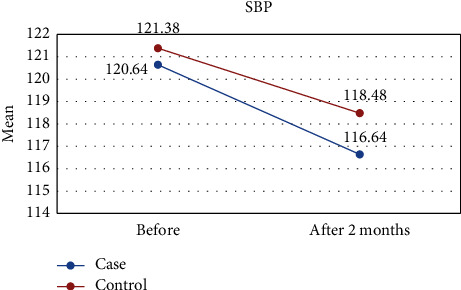
Effect of lycopene supplementation on SBP levels.

**Figure 8:**
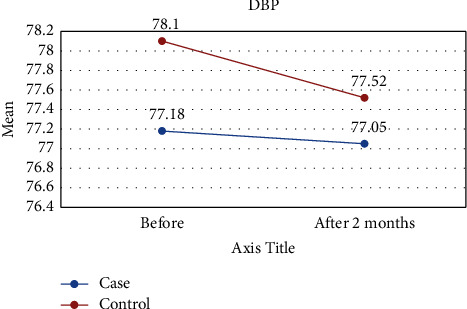
Effect of lycopene supplementation on DBP levels.

Table 1 shows the results of this study. The results showed that the values of all variables before the intervention in both groups were almost the same and did not differ significantly. However, two months after the intervention, the amount of TG and FMD was significantly different between the two groups, so in the intervention group, the mean of TG was lower (Figure2) and the mean of FMD was higher (Figure 3) than those of the control group. However, for other variables including LDL-C, HDL-C, TC, DBP, and SBP, there were no significant differences between the intervention and control groups (Figures 4–8).

The results of the Kruskal–Wallis test for intergroup analysis to evaluate the difference between before and after the intervention in each group separately showed that for variables TG, TC, LDL-C, and HDL-C in both intervention and control groups, the mean of these variables improved significantly 2 months after the intervention. Only in the intervention group, there was a significant difference in SBP and FMD variables before and after the intervention. In both cases, DBP did not differ significantly between the intervention and control groups.

## 4. Discussion

Many clinical trials and review studies showed that tomato and lycopene have health benefits [19–25]; however, only a few of them mainly investigated the effect of lycopene supplement intake on CVD risk factors in HF patients. Our study showed that lycopene supplementation in HF subjects improves TG and FMD compared to the control but no other variables (TC, LDL-C, HDL-C, SBP, and DBP). Also, only SBP and FMD showed intragroup improvement in the intervention group.

### 4.1. Blood Pressure

Our results, however, showed no positive effect for lycopene on SBP and DBP between groups at the 25 mg daily dose, but SBP decreased preintervention and postintervention. Our results were consistent with findings of the previous study that lycopene has positive effects on SBP at 10–15 mg of lycopene/day, especially in hypertensive people [26]. A meta-analysis also reported that a lycopene supplement of >12 mg/day could successfully lower SBP, especially in Asians and individuals with higher baseline SBP [27]. Their results, like ours, showed that lycopene has no significant positive effect on DBP. Since the HF patient is on several other HF medications, in addition to angiotensin-converting-enzyme inhibitors (ACEI) and *β*-blockers, including angiotensin receptor blockers, aldosterone-antagonists, hydralazine, and isosorbide, hypotension has been reported as adverse events in 6–8% of patients [28]. Therefore, the antihypertensive aspects of lycopene may vary in these special groups of CVD patients.

### 4.2. Flow-Mediated Dilatation (FMD)

FMD is used to assess endothelial function in clinical investigations and describes the vasodilatory response of the artery to elevations in blood flow-associated shear stress [29]. Our results showed that FMD was significantly improved between groups and also in intraintervention groups. Armoza et al. have shown that in an in vitro study, lycopene improves basal endothelial function as measured by increased release of NO and decreased expression of endothelin-1 (ET-1) [30]. An animal study also proved the positive effect of lycopene on endothelial function. Cheng et al. carried out a meta-analysis including 233 participants and assessed the impact of tomato supplementation on FMD in marathon runners, healthy postmenopausal women, and healthy subjects. They observed that the use of lycopene significantly improved FMD by 2.53%. Our findings also confirm the effectiveness of lycopene supplementation in improving FMD in ischemic HFrEF patients [31].

### 4.3. Lipid Profile

According to our results, only TG improved in the intervention group following lycopene supplementations at 25 mg daily compared to the control, and other lipid profile components including LDL-C, HDL-C, and TC did not improve significantly. However, it has been reported previously that lycopene administered at doses of 25 mg daily reduces LDL-C by around 10%, which is comparable to the impact of low-dose statins in patients with mildly increased cholesterol levels [5]. Our results in line with findings of the study by another study reported that the lycopene-containing regimen did not affect the plasma lipid profile compared to the control diet [6]. An animal study showed that dietary lycopene supplementation significantly decreased the levels of plasma, TC, TG, and LDL-C in the feedlot lamb [32]. A meta-analysis on the serum lipid profile also found a significant cholesterol-lowering effect of lycopene on TC and LDL-C in humans for dosages of ≥25 mg daily [33]. In a meta-analysis involving 12 test groups and 781 subjects, a significant increase in HDL-C levels was reported in the lycopene group compared to the control group, and there was no significant difference in TG levels between the groups [24]. However, no other meta-analysis showed a significant increase in HDL-C, except for a single subgroup meta-analysis [33–35]. Another study concluded that lycopene supplementation for CV risk factors supports the idea that increasing these foods' intake improves blood lipids [35]. Another study on CVD patients using lycopene from cooked tomatoes similar to our study reported no significant improvement in LDL-C, TC, and HDL-C levels [36]; however, unlike them [34, 36] who did not see a decrease in TG levels, we saw a significant decrease in TG levels between groups.

### 4.4. Mechanisms

Lycopene has a chemical formula of C40H56 with 13 double bonds altogether, 11 of which are conjugated, and therefore, it is regarded as one of the strongest antioxidants because of its structure. Due to the high level of reactivity between the long polyene chain and free radicals, lycopene is a very potent antioxidant that helps reduce ROS and eliminate singlet oxygen [37, 38]. Lycopene is well-known to act on additional free radicals such hydrogen peroxide (H_2_O_2_), nitrogen dioxide (NO_2_), and hydroxyl radicals in addition to quenching singlet molecular oxygen. Lycopene is thought to strengthen the cellular antioxidant defense system by recovering nonenzymatic antioxidants from their radicals, such as vitamins E and C [39]. The possible antioxidant capabilities of lycopene are thought to be the cause of its cardioprotective effects. Also, by blocking the angiotensin-converting enzyme (ACE), lycopene reduces the oxidative stress imposed on by angiotensin II and, as a result, indirectly increases the synthesis of NO in the endothelium [40]. It has been shown that lycopene suppresses the production of reactive oxygen species and induces the expression of heme oxygenase-1 (HO-1) in human endothelial cells, lowering ET-1 gene expression. As a result, it may help prevent endothelial dysfunction by encouraging direct antioxidative actions and increasing the expression of several genes [41].

#### 4.4.1. Strengths

The fact that our investigation was done as a randomized controlled clinical trial was its primary strength. Our participants were CVD patients with ischemic heart failure and increased ejection fraction. Instead of tomato, only lycopene as the main ingredient of tomato was studied.

#### 4.4.2. Limitations and Recommendations

All patients in this study were from one center and one city. Also, we did not use different doses of this supplement, so we cannot conclude the effect and role of the dose consumed in its effectiveness. Further research using different doses of lycopene supplementation on cardiovascular parameters and markers of inflammation and oxidation in ischemic HF patients is highly recommended.

## 5. Conclusions

It can be concluded that supplementing with lycopene can enhance endothelial function and reduce the TG levels in ischemic HFrEF patients. However, it had no positive effect on BP, TC, LDL-C, or HDL-C. Due to the disparity between the lipid profile and the effect of lycopene on BP in individuals with HF, more clinical trials are required to achieve validated conclusions.

## Figures and Tables

**Table 1:** Summary of the results.

Variables		Case (*N* = 22) (mean ± Sd)	Control (*N* = 21) (mean ± Sd)	*P* value^I^
TG	Before	247.36 ± 16.80	247.67 ± 17.77	0.874
	After 2 months	**219.27** **±** **19.48**	**234.24** **±** **17.48**	**0.002**
	*P* value^II^	**<0.001**	**<0.001**	

TC	Before	205.32 ± 29.31	201.43 ± 14.46	0.836
	After 2 months	181.09 ± 31.21	175.95 ± 24.38	0.301
	*P* value^II^	<0.001	<0.001	

HDL-C	Before	42.86 ± 7.16	43.43 ± 6.80	0.788
	After 2 months	45.64 ± 8.35	46.43 ± 8.03	0.733
	*P* value^II^	0.002	0.001	

LDL-C	Before	76.50 ± 15.17	73.86 ± 14.98	0.519
	After 2 months	61.77 ± 15.64	66.76 ± 14.94	0.325
	*P* value^II^	<0.001	<0.001	

SBP	Before	120.64 ± 16.19	121.38 ± 16.05	0.893
	After 2 months	116.64 ± 17.12	118.48 ± 14.46	0.635
	*P* value^II^	0.002	0.225	

DBP	Before	77.18 ± 8.51	78.10 ± 9.28	0.806
	After 2 months	77.05 ± 8.23	77.52 ± 9.23	0.815
	*P* value^II^	0.702	0.725	

FMD	Before	2.36 ± 1.26	2.62 ± 1.83	0.784
	After 2 months	**5.68** **±** **1.81**	**2.95** **±** **1.50**	**<0.001**
	*P* value^II^	**<0.001**	**0.115**	

I: Mann–Whitney test; II: Kruskal–Wallis test.

## Data Availability

The Chamran Hospital in Isfahan, Iran, has a collection of data that supports the conclusions in this investigation; however, access to these data is restricted because they are used under permission for the current study and are not publicly available. The data are available upon reasonable request from the corresponding author with Chamran Hospital's permission.
